# Preparation and Characterization of *Pistacia khinjuk* Gum Nanoparticles Using Response Surface Method: Evaluation of Its Anti-Bacterial Performance and Cytotoxicity

**DOI:** 10.15171/apb.2017.020

**Published:** 2017-04-13

**Authors:** Ali Fattahi, Tahereh Sakvand, Marziyeh Hajialyani, Behzad Shahbazi, Mohammad Shakiba, Ahmad Tajehmiri, Ebrahim Shakiba

**Affiliations:** ^1^Medical Biology Research Center, Kermanshah University of Medical Sciences, Kermanshah, Iran.; ^2^Nano Drug Delivery Research Center, Faculty of Pharmacy, Kermanshah University of Medical Sciences, Kermanshah, Iran.; ^3^Pharmaceutical Sciences Research Center, Faculty of Pharmacy, Kermanshah University of Medical Sciences, Kermanshah, Iran.; ^4^Department of Medicinal plants, Kermanshah Jahade-Daneshgahi, Institute of Higher Education, Kermanshah, Iran.; ^5^Student Research Committee, Kermanshah University of Medical Sciences, Kermanshah, Iran.; ^6^Department of Biochemistry, Medical School, Kermanshah University of Medical Sciences, Kermanshah, Iran.

**Keywords:** Antibacterial activity, Cytotoxicity, Nanoparticle, Pistacia Khinjuk, Response Surface Model

## Abstract

***Purpose:*** This study aims to prepare a novel, natural nanoparticle (NP) as a drug carrier, which also has inherent therapeutic effects.

***Methods:*** Pistacia khinjuk gum NPs were prepared and Response surface methodology (RSM) was used for statistical analysis of data and optimizing the size of NPs.

***Results:*** NPs were in the range of 75.85–241.3 nm. The optimization study was carried out, and an optimized size (70.86nm) was obtained using DMSO as a solvent. The volume of the organic phase was 111.25µl, and the concentration of gum was 1% w/v. The cell viability assay was performed on the pure gum and NPs toward β-TC_3_, MCF7, and HT29 cell lines. It was observed that NPs have higher cytotoxic activity in comparison with pure gum, and that the IC_50_value was achieved at 1% of NPs in β-TC3 cells. The obtained NPs demonstrated antibacterial activity against two bacterial strains (Pseudomonas aeruginosa and Staphylococcus aureus).

***Conclusion:*** Altogether, according to the obtained results, these NPs with inherent cytotoxicity and antibacterial activity are an attractive carrier for drug delivery.

## Introduction

*Pistacia khinjuk* is one of the major Pistacia species that grows in some of Mediterranean countries (they have especially been widely distributed in the *Zagrossian* region of Iran) and classified into the *Anacardiaceae* family. Different parts of the plant, including resin, leaf, bark, fruit, and aerial parts, can be used as traditional medicine.^1^ They have been used for a long time as useful remedies for the treatment and prevention of different kinds of diseases such as asthma and stomach discomfort, throat infections, burns, nausea, eczema, vomiting, and toothaches;^2-5^ specifically, the gum resin has exhibited wound healing activity that could be used for the treatment of brain and gastrointestinal disturbances.^2^ Due to the literature reports, *p. khinjuk* has exhibited inherent anti-inflammatory, antileishmanial, antipyretic, antioxidant, antitumor, antiviral, antiasthmatic, and antimicrobial properties.^2,6,7^ In addition to these common medical applications*, p.khinjuk* gum could be a candidate as a new natural biopolymer for drug delivery systems.

According to the literature review, the fabrication of p.khinjukgum NPs and using them as a novel delivery vehicle has not been previously investigated. Due to the numerous desirable characteristics and advantages of *p.khinjuk* gum in therapeutic objectives, the fabrication of *p.khinjuk* gum NPs as drug carriers was investigated in this study. The obtained data was statistically analyzed using RSM, which combines statistical and mathematical techniques to fit experimental data to the model for optimization processes.^8^

## Materials and Methods

### Materials

*P.khinjuk* gum was collected from the exudates of the trunk of *p.khinjuk* tree (from Oshtoran-Kooh Mountain, in Azna, Lorestan province, Iran) in July 2014. The organic solvents (acetone, ethanol and DMSO) were purchased from Merck (Germany). Trypsin-ethylenediamine tetra acetic acid (EDTA) was supplied from Ben Yakhte, Iran. DMEM (Dulbecco's Modified Eagle's Medium) and Roswell Park Memorial Institute medium (RPMI) were procured from Gibco, Scotland. Thiazolyl blue (MTT) was purchased from Merck, Germany.All of the other compounds were of analytical grade from Merck.

### Preparation of NPs

The organic solutions were prepared using three organic solvents (ethanol, acetone, and DMSO). *P.khinjuk* gum with three different amounts of 0.1, 0.55, and 1g, was dissolved in 100 ml of each organic solvent to prepare three different concentrations of *p.khinjuk* gum (0.1%, 0.55%, and 1%w/v).Then 100, 500, and 1000 µl of each stock solution was added dropwise to 10 ml of distilled water under stirring. The resulting solutions were stirred for 1 h at 2500 rpm and at room temperature.

### Experimental design

In this study, optimization of the size of NPs was carried out according to the Central Composite Face-Centered Design (CCFD) and using Design-Expert software (Version 8.0.7.1, statEase, Inc., USA).

### Characterization of NPs

The size of fabricated particles was assessed and analyzed by Zetasizer (Nano-ZS, Malvern, UK) using dynamic light scattering (DLS).

The morphology and structure of NPs were observed by a transmission election microscope (TEM, Zeiss-EM10C, 80 KV, Germany).

### Cell viability assay

In this study, β-TC_3_ (a mouse beta pancreatic cell line), was purchased from Iran genetic resources center. MCF7 (a human breast cancer cell line) and HT29 (a human colon adenocarcinoma cell line) were purchased from Pasteur Institute of Iran. The thiazolyl blue assay has been used in experiments for the assessment of cell viability. Briefly, cells were seeded at density of 5 × 10^4^ cells/ml in 96-well tissue culture. After 24 h, cells were incubated with increasing concentrations of pure gum and NPs (1%, 0.5%, 0.25%, and 0.125% w/v) for 48 h separately. Then the MTT assay was performed to measure the cell viability according to previous study.^9^

### Antibacterial test

*Pseudomonas aeruginosa* (ATCC27853), as a standard strain, and *Staphylococcus aureus*, as a clinicallyisolated strain, in the Microbiology Laboratory of Imam Khomeini Hospital (Kermanshah, Iran) were used in this study.

Serial dilutions (1.5%, 1.3%, 1.1%, 0.9%, and 0.7%) of the NPs were made in Mueller-Hinton Broth containing 5% DMSO for bacteria, in 96-well micro titer plates. 20 µl of fresh microbial suspensions was prepared from overnight grown cultures containing 1.5 × 10^8^ organisms/ml and were added to each well. The final volume of culture was 200 µl per each well.

Ampicillin and water were used as positive and negative controls, respectively. The MTT assay was performed to assess the (MIC) and MBC of the extract of NPs using MTT solution with concentration of 5.0 mg/ml.

## Results and Discussion

### Response Surface Model

Table 1 shows the actual form of factors and the experimental size of NPs, as the response. Based on the data, the range of responses was found from 75.85–241.3 nm. The response function was fitted by a quadratic polynomial model.

**Table 1:** Factors in actual form, and experimental size data

**Organic phase volume (μl)**	**Concentration of gum (w/v%)**	**Solvent type**	**Size(nm)**
1000	0.55	Ethanol	159
1000	0.55	Acetone	195.6
1000	0.55	DMSO	118.2
550	0.55	Acetone	189.66
550	0.55	DMSO	114.6
550	0.55	Acetone	183.3
550	0.55	Ethanol	117.1
550	0.55	Ethanol	116.5
550	0.55	Ethanol	113.5
550	0.55	Acetone	149.66
550	0.55	DMSO	105.9
550	0.55	Acetone	164.1
550	0.55	DMSO	100.9
550	0.55	DMSO	98.02
550	0.55	Ethanol	138
550	0.55	DMSO	107.3
550	0.55	Ethanol	134.1
550	0.55	Acetone	168.4
100	0.55	DMSO	78.86
100	0.55	Acetone	135.6
100	0.55	Ethanol	107.1
1000	1	DMSO	102
1000	1	Ethanol	186.6
1000	1	Acetone	241.3
550	1	DMSO	87.52
550	1	Acetone	202.3
550	1	Ethanol	164.5
100	1	Acetone	186.6
100	1	DMSO	97.2
100	0.1	Ethanol	144.8
1000	0.1	DMSO	155.2
1000	0.1	Acetone	140
1000	0.1	Ethanol	118
550	0.1	DMSO	114.5
550	0.1	Ethanol	82.96
550	0.1	Acetone	106.9
100	0.1	Acetone	96.32
100	0.1	DMSO	104.5
100	0.1	Ethanol	75.853

To find the best model correlating the response to process variables the analysis of variance (ANOVA) by calculating F-value was employed. It is important to note that the p-values< 0.05 indicate a better significance of model terms. The lack of fit F-value of 0.7007 revealed that the lack of fit is not significant in response, which indicates low error and accuracy of the model (Table 2).

According to the experimental results and using RSM, the response function was fitted by a quadratic polynomial equation. This equation is given as follows in terms of coded factors:

Z average = +50.85+0.05828A+89.116B −0.0145 AB (ethanol) (1)

Z average = +71.027+0.0666A+114.2620B − 0.0145 AB (acetone) (2)

Z average = +100.63+0.0431A − 24.427B− 0.0145 AB (DMSO) (3)

Where Z average is the average of size, A is the volume of the organic phase, and B is the percentage of gum in the solution.

The regression analysis shows that all the linear coefficients of the independent variables, and also the interaction of the volume of the organic phase and the percentage of gum in the solution, are significant (where p<0.05).

The coefficient of determination (R^2^), adjusted R^2^, and predicted R^2^ of the model were found 0.93, 0.92, and 0.90, respectively.

**Table 2:** Analysis of Variance

**Source**	**Sum of Squares**	**D**_f_	**Mean Square**	**F-Value**	**p-value**	**Prob> F**
**Model**	55183.41	6.00	9197.24	76.88	< 0.0001	Significant
**A-Organic solvent volume**	8409.62	1.00	8409.62	70.30	< 0.0001	
**B-Concentration of gum**	9734.17	1.00	9734.17	81.37	< 0.0001	
**C-Type of solvent**	23772.23	2.00	11886.12	99.36	< 0.0001	
**BC**	13267.39	2.00	6633.70	55.45	< 0.0001	
**Residual**	3827.95	32.00	119.62			
**Lack of Fit**	2143.51	20.00	107.18	0.76	0.7132	not significant
**Pure Error**	1684.44	12.00	140.37			
**Cor Total**	59011.36	38.00				

**Figure 1:**
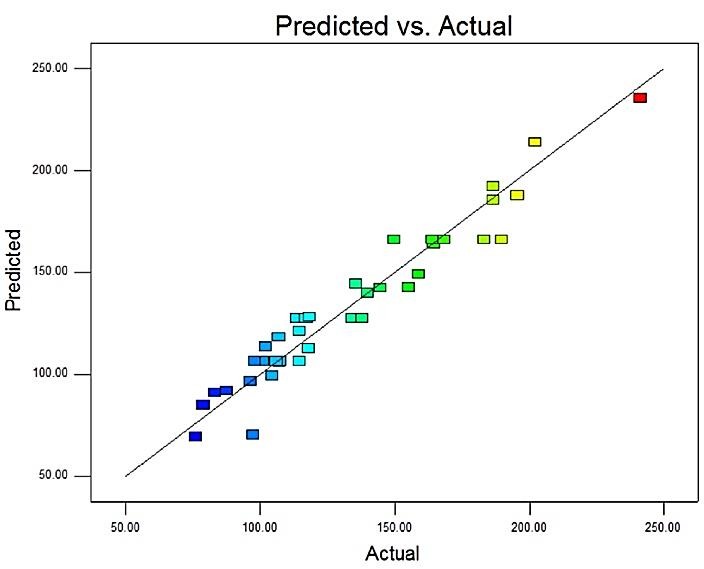
Predicted versus actual values of Zaverage for RSM mode

Figure 1 shows the distributed plot of the predicted amounts versus the actual amounts for the size of NPs. The closer the points are to the 45 degree line, the better the estimations of the RSM model. Based on this plot, the model could appropriately fit the data.

**Figure 2:**
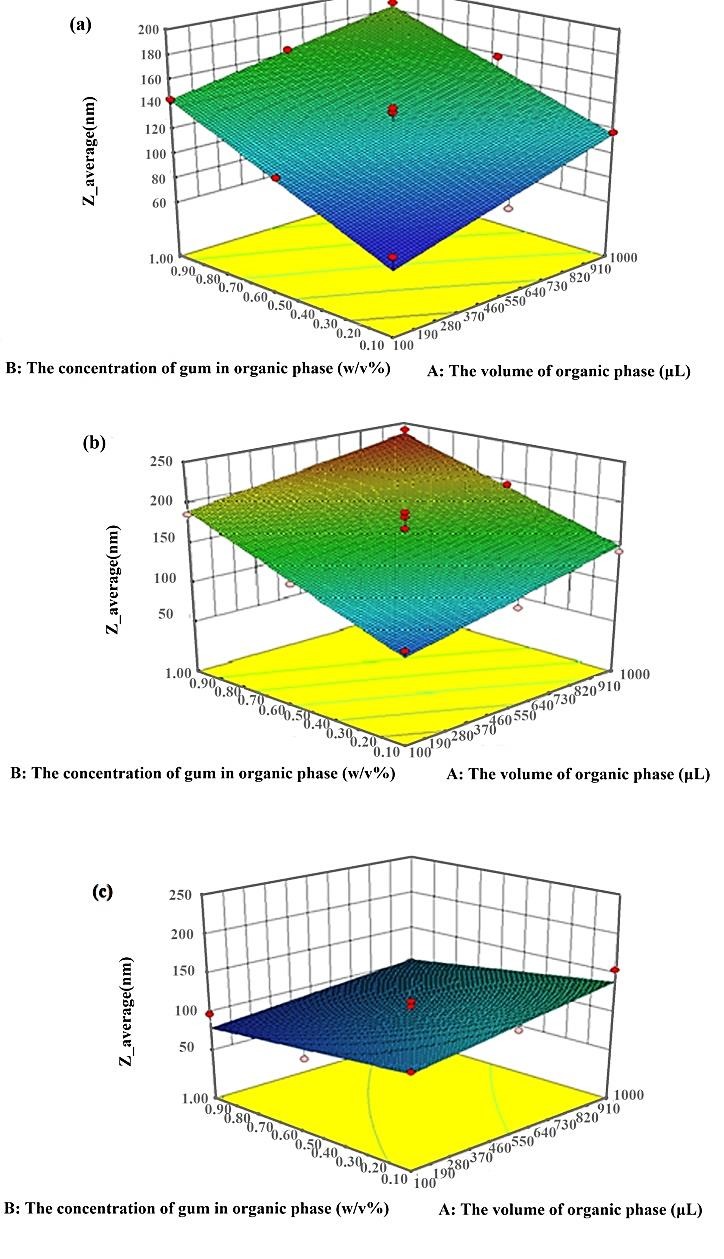
The effect of volume of organic phase and the concentration of gum in organic solution on Z average of NPs obtained by a) ethanol; b) acetone; and c) DMSO as organic solvent.

Figure 2 shows the response surface 3D plots exhibiting the effects of interactions between the volume of the organic phase and the concentration of gum in the organic phase.

### Effect of process parameters on the size of NPs

The size of the fabricated NPs was measured and the results are tabulated in Table 1. In the presence of ethanol and acetone, increasing the gum concentration causes fabrication of larger NPs. But in the presence of DMSO, the results were the inverse, and increasing the concentration of gum resulted in the reduction in the size of NPs. As the concentration of gum in the organic phase increases, the size of fabricated NPs increases due to an increase in the viscosity of organic solution and the hindering of the diffusion of solvents to water. Diminution in viscosity leads to the facilitation of solvent diffusion to the outer aqueous solution and consequently, production of smaller particles.^10^ This trend was conversely when DMSO was used as the organic solvent. This incongruity may arise from possible interactions between *p.khinjuk* gum and DMSO molecules.

The results also revealed that, at constant volume of aqueous solution, increasing the volume of the organic phase (increasing the volume ratio of the organic to aqueous solutions) results in an increase in the size of fabricated NPs. Decreasing the ratio of organic to aqueous solutions can improve the diffusion of organic solvent, and it increases the distribution efficiency of the organic phase into the external phase, leading to formation of smaller NPs. This reason has been evoked in the similar studies.^11^

The size of NPs using different solvents is exhibited in Table 1. The comparison revealed that using DMSO as the solvent, results in smaller NPs at the highest concentration of gum. DMSO was chosen as the most suitable solvent due to the production of smaller particles. It is worthmentioning that the selection of organic solvent could be directly affected by individual key parameters, such as a solvent dielectric constant and the affinity of the solvent for water. The miscibility of solvents in water is an important parameter that should be considered. The higher miscibility of the solvent in water causes the higher rate of diffusion into the aqueous phase, and consequently, the production of smaller NPs. The miscibility of solvents in water could be evaluated regarding the mutual solubility parameter (Δδ), The smaller Δδ indicates the higher affinity.The Δδ has been evaluated 27, 28.7, and 34.4 MPa^1/2^for ethanol, DMSO, and acetone, respectively.^12^

### Optimization and model validation

To determine the optimum condition for the lowest Z average, some solutions would be suggested by the software. Based on these results, the optimum size of NPs was obtained using DMSO as the organic solvent (using 111.25 µl of organic phase and 1% of gum). For model validation, experiments were performed by using the aforementioned optimum conditions. The experimental response for optimized NP size was 73.18 nm, and the prediction error was found 0.0327 (<0.05), which confirms the validity of model in optimizing the size of NPs.

### Morphology of NPs 

**Figure 3:**
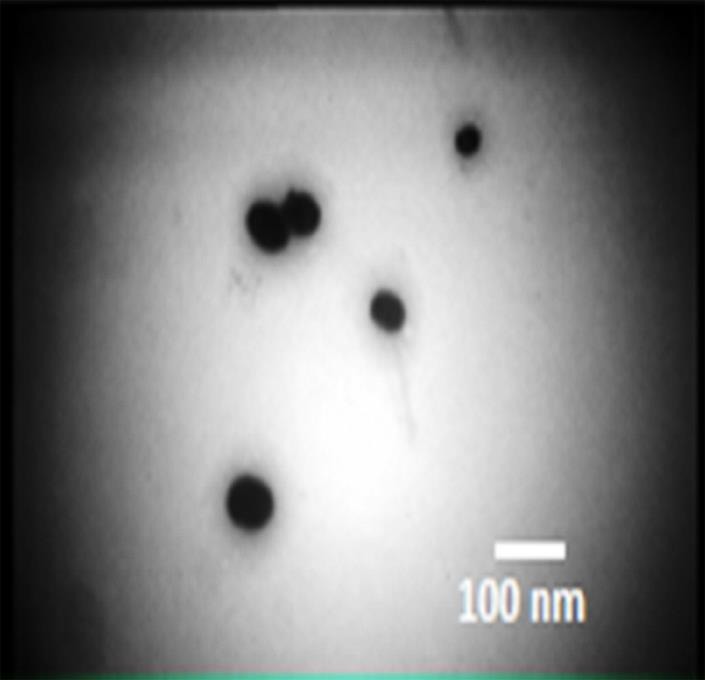
TEM of the suspension of NPs in DMSO with the concentration of 1% w/v.

The TEM result of the suspension of NPs (at the concentration of 1% w/v using DMSO as organic phase) is visualized in Figure 3. The polydispersity index of these NPs was measured using Zetasizer. According to the TEM observation, particles were found to be relatively spherical, and the size of the obtained NPs was less than 100 nm. The obtained NPs were monodisperse, and the polydispersity of NPs was found 0.07, which confirms the narrow size distribution of NPs. The measured values resulted from TEM was comparable with the DLS results.

### The cytotoxicity test

**Figure 4:**
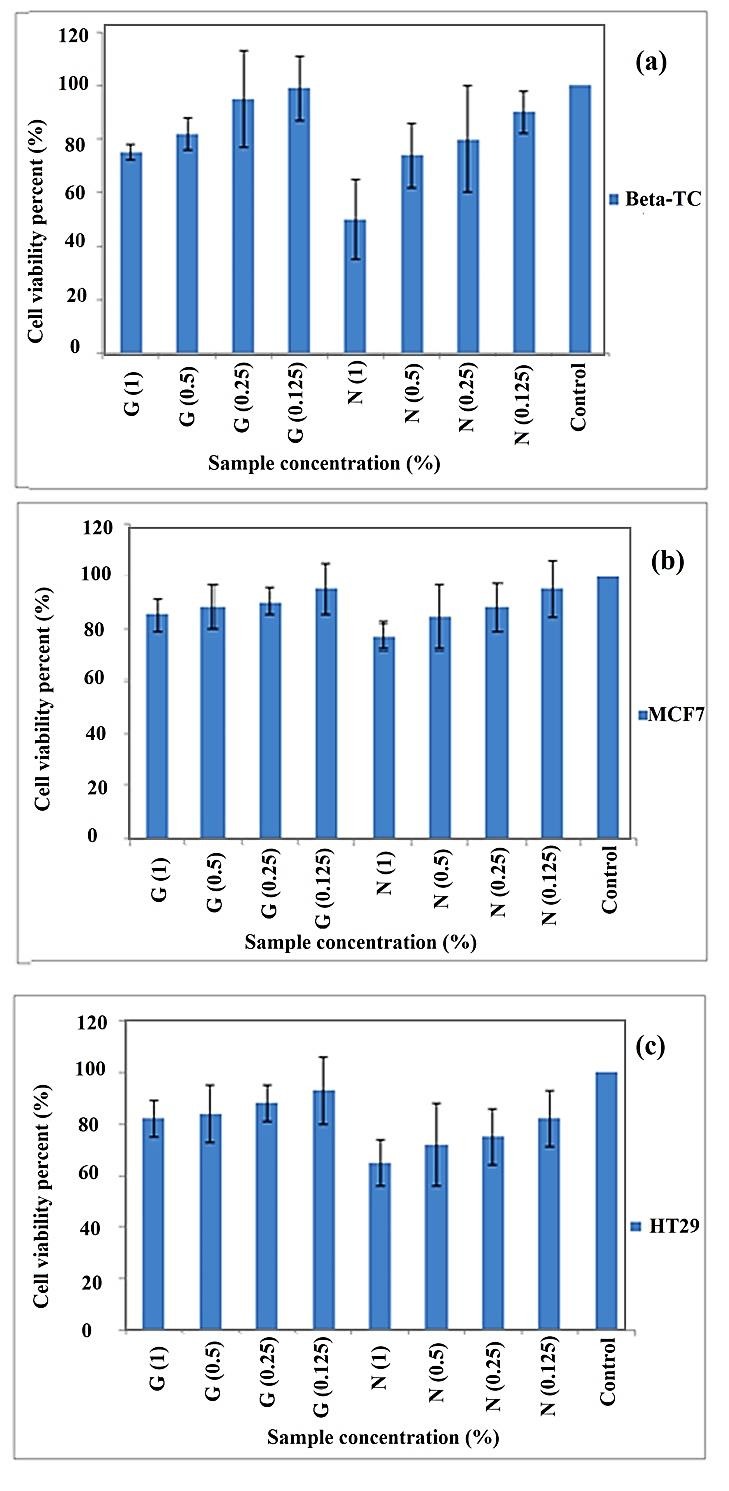
Cell viability of pure gum and NP on a) β-TC_3_; b) MCF7; and c) HT29 cell lines (*G refers to Gum and N refers to NP).

The MTT results indicated that NPs have higher cytotoxic activity compare to pure gum in all three cell lines (β-TC_3_, MCF7, and HT29), while NPs and gum exhibited the relatively highest cytotoxic activity towards β-TC_3_ (Figure 4). The IC_50_value was achieved at 1% of NPs in β-TC_3_. The results revealed that the cytotoxicity of pure gum and NPs does not reach IC_50_ in HT29 and MCF7 cell line. The cytotoxic activity of NPs and pure gum can be attributed to the high content of terpenes in *p.khinjuk* gum structure.^13^ The Hedgehog (Hh) signaling pathway is a critical element regulating cellular growth and organizing differentiation during embryonic development, which plays a significant role in different types of cancer.^14,15^ Terpenes possess the ability to affect and target the Hh pathways and have been used for treating cancer. This pathway has been frequently discussed in the treatment of pancreatic cancer and has a significant effect on the treatment of this cancer, but it has no significant effect on HT29; the effect of this pathway on colon cancer is not clear. The Hh pathway also affects breast cancer, but there have some inconsistent reports on the effect of Hh on the MCF7 cell line. While this pathway has been found effective on the MCF7 cell line, its growth could not be inhibited by cyclopamine.^15,16^ There is no clear report for the mechanism of Hh activation in breast cancer, and the mechanism of Hh effect on MCF7 is not clear yet.

The higher cytotoxic activity of NPs could be due to the small size of the particles and advantageous for nano-sized particles. Furthermore, it could be noted that pure gum is insoluble in water, while NPs could disperse homogenously in water and are able to transport more into cells, thus achieving lower cell viability and showing greater cytotoxicity compared to gum.

### Measurement of antibacterial activity

Table 3 shows the results of antibacterial activity of NPs towards both performed bacterial strains.The inhibition activity of the samples can be attributed to the fact that the major constituent of gum is α-pinene. The antimicrobial activity of α-pinene has been reported in the literature.^17^

## Conclusion

In the current study, *p.khinjuk* gum NPs, were prepared as novel drug carriers and the size of particles could be altered with different affecting parameters. The optimization was performed, and the optimum size was achieved using 111.25µl of DMSO, and 1% (w/v) of gum, with the NP size of 70.86 nm (obtained by model). The effect of experimental parameters on the size of NPs was obtained by fitting experimental data with a quadratic equation with a prediction error less than 0.05. The obtained NPs were found monodisperse and spherical, according to the DLS and TEM results. The obtained NPs had higher cytotoxic activity in comparison with pure gum and its IC_50_ value was achieved at 1% of NPs in β-TC_3_. These NPs also possess inherent antibacterial activity and could be good candidates for treatment and as carriers in drug delivery systems.

**Table 3:** The antibacterial activity of NPs.

**Bacterial Strains**	**Concentration**				
	**0.7%**	**0.9%**	**1.1%**	**1.3%**	**1.5%**
*Pseudomonas aeruginosa*	+	+	MIC	+	MBC
*staphylococcus aureus*	+	+	MIC	MBC	_

## Acknowledgments

The authors would like to acknowledge the Research Council of Kermanshah University of Medical Sciences for supporting this work (Research grant No.94219).

## Ethical Issues

Not applicable.

## Conflict of Interest

The authors declare no conflict of interests.
